# Supplementary material for: Functional equivalence of genome sequencing analysis pipelines enables harmonized variant calling across human genetics projects
Source: Nat Commun. 2018 Oct 2;9:4038. doi: 10.1038/s41467-018-06159-4 (PMC6168605; doi:10.1038/s41467-018-06159-4)
Supplement: Supplementary file 1 — Supplementary Information [file 41467_2018_6159_MOESM1_ESM.pdf]

Supplementary Figures

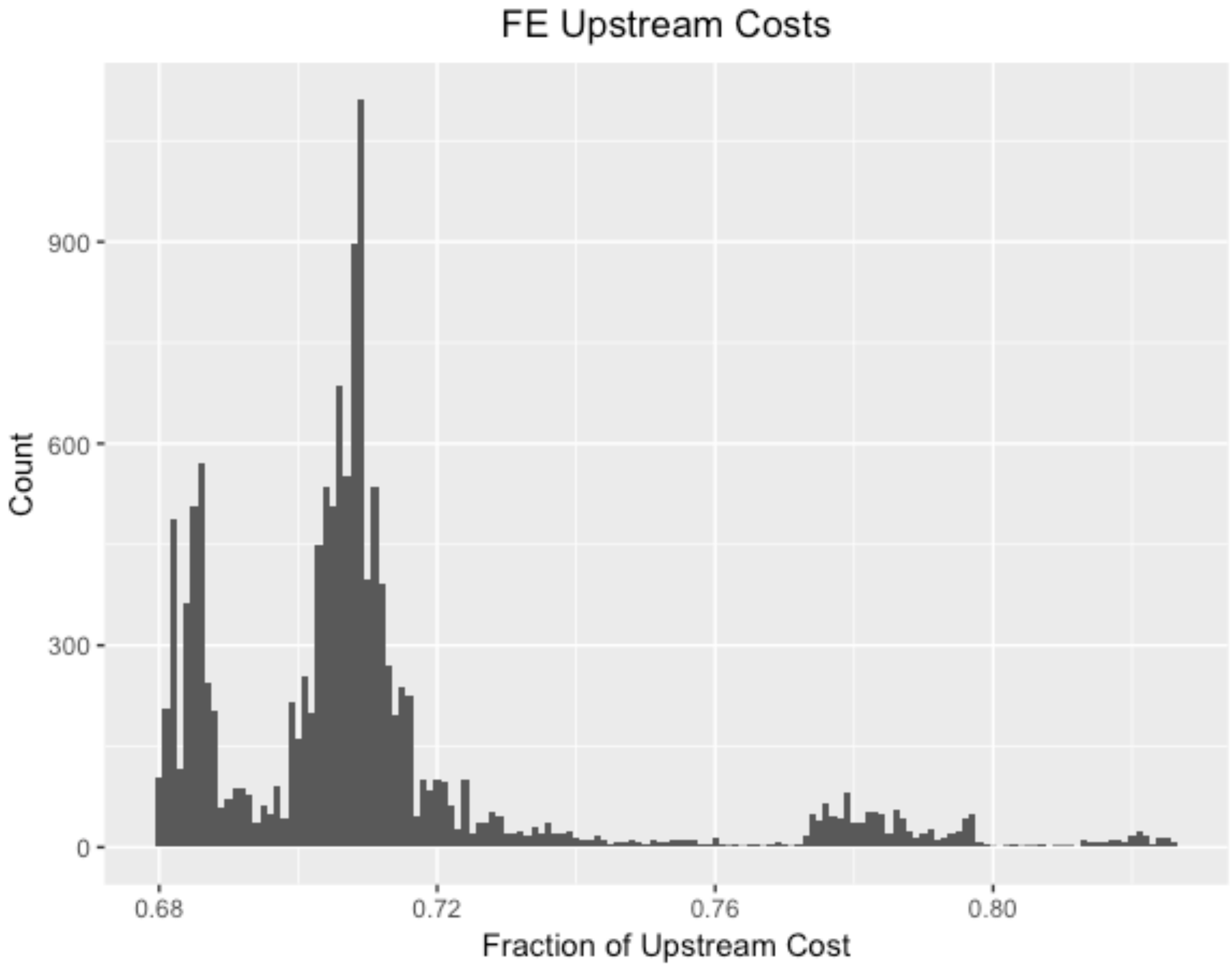

Supplementary Figure 1. Functional equivalence upstream cost. The steps upstream of variant calling (alignment, data processing, and qc) accounted for 68%-83% of the total per-sample costs in 13,704 successful runs of the Broad Institute's harmonized pipeline.

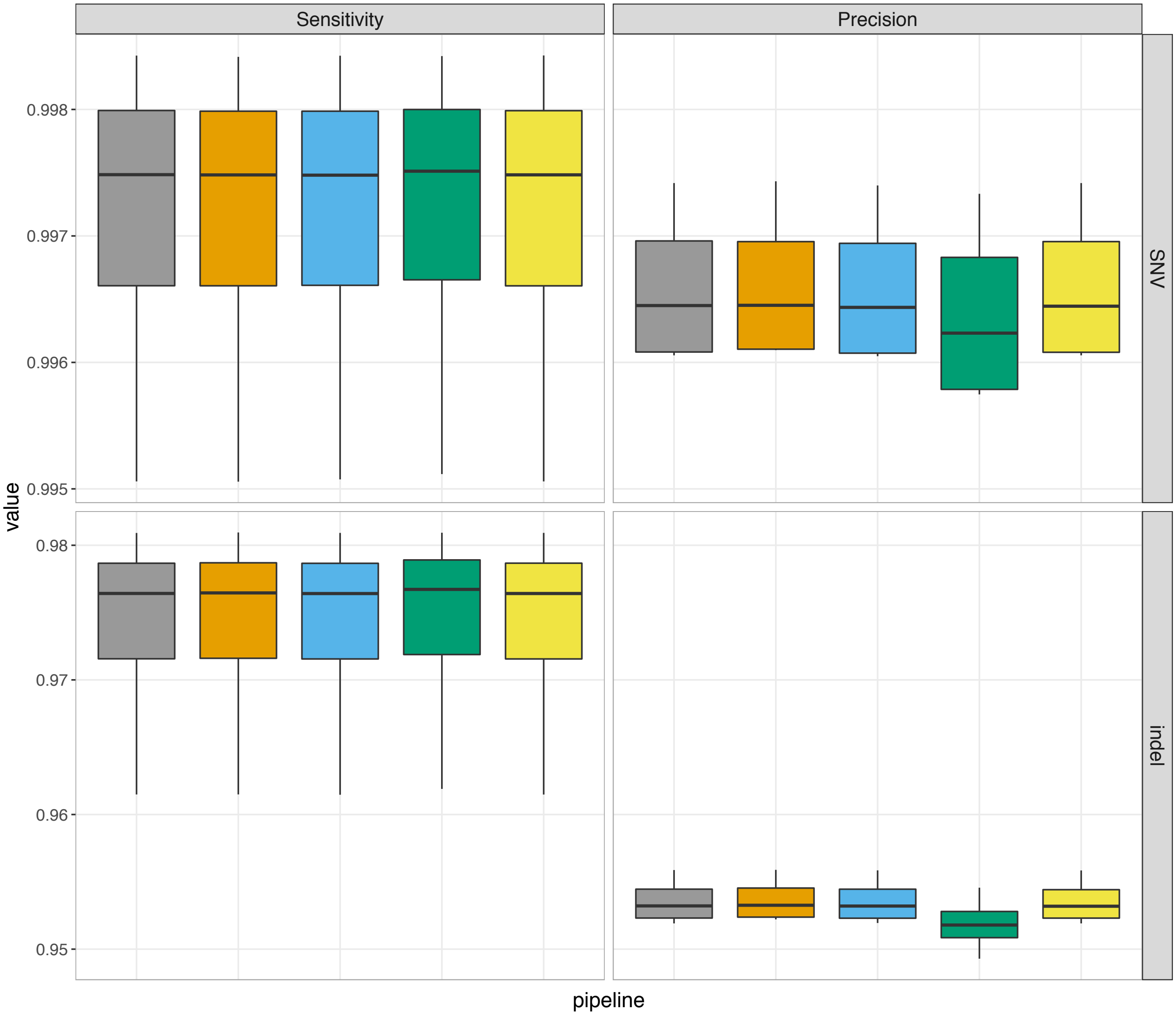

Supplementary Figure 2. Comparison to gold standard variants. Sensitivity and precision to the GiaB gold standard variants were very similar across pipelines for all four NA12878 replicates, although one center with different pipeline components is slightly more sensitive and less precise. The center line is the median, the upper and lower hinges are the first and third quartiles, and the whiskers extend to the largest/smallest values no further than 1.5 * inter-quartile range from the hinge.

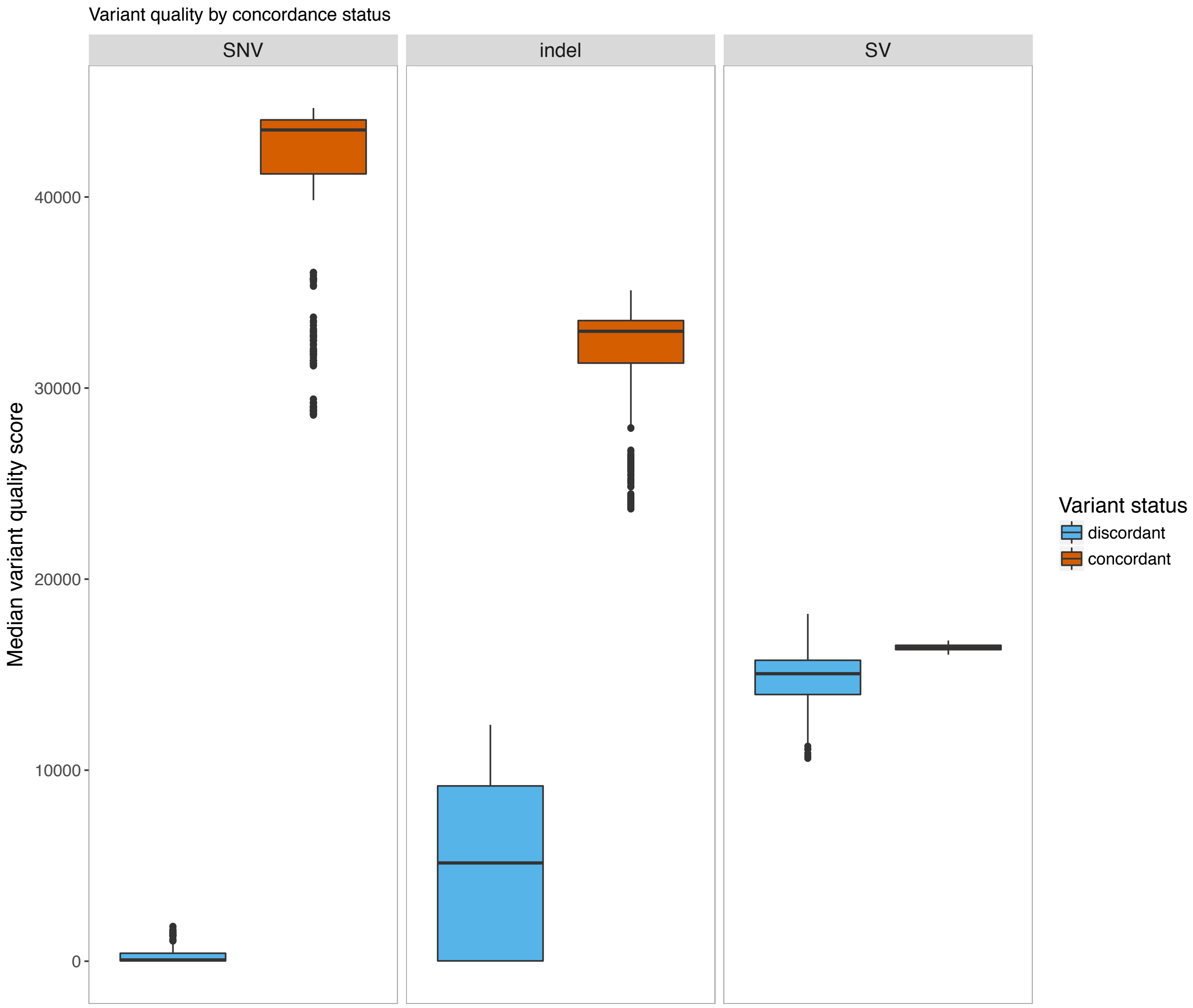

Supplementary Figure 3. Variant quality score by concordance status. The median variant quality score (QUAL field from the GATK VCF; MSQ INFO field from the LUMPY SV VCF) was calculated for each sample, with variants partitioned by their status in each pairwise pipeline comparison. The center line is the median, the upper and lower hinges are the first and third quartiles, and the whiskers extend to the largest/smallest values no further than 1.5 * inter-quartile range from the hinge.

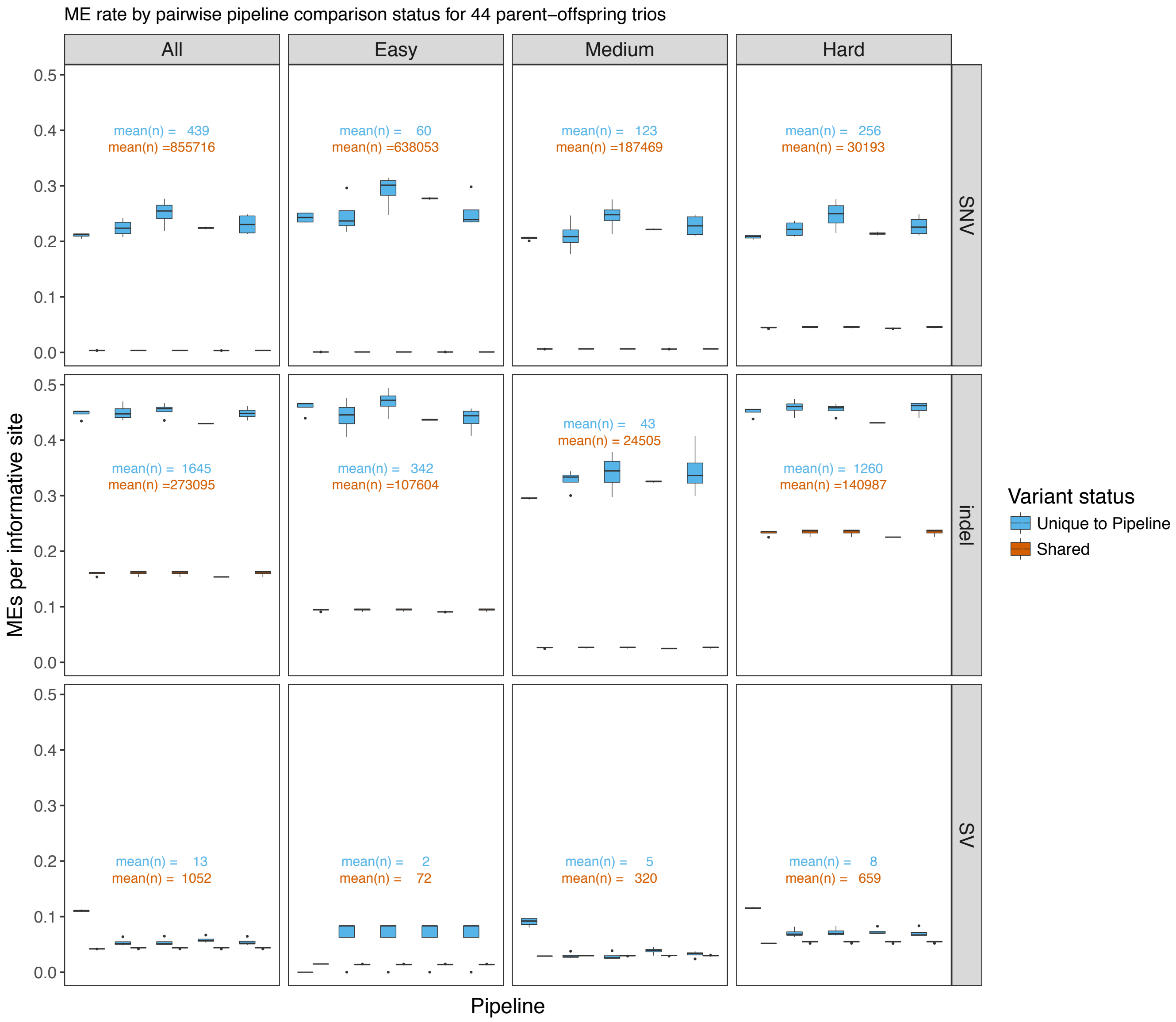

Supplementary Figure 4. Mendelian error rate by concordance status per pipeline. The rate of Mendelian error for each of 44 parent-offspring trios was calculated for variants shared between two pipelines as well as variants unique to one pipeline. The error rate was determined using informative sites only. In most variant types and genomic regions, variants unique to each pipeline show similar error rates, indicating that no pipeline is introducing variant calling errors or improvements in a biased way. The exception is SVs, where unique variants from one pipeline have a higher error rate than other pipelines; but, note that this is caused by a tiny number of discordant calls. The center line is the median, the upper and lower hinges are the first and third quartiles,

and the whiskers extend to the largest/smallest values no further than 1.5 * inter-quartile range from the hinge.

Supplementary Tables

Supplementary Table 1. Select alignment statistics for NA19431, post-harmonization.

Center	Center 1	Center 2	Center 3	Center 4	Center 5
## Alignment Statistics					
Yield_Reads	841,563,833	841,496,939	841,496,939	841,496,939	841,496,939
Unmapped_Reads	14,505,992	12,605,618	12,605,618	12,605,618	12,605,618
Duplicate_Reads_PCT	6.02	6.02	6.02	6.02	6.02
Q20_Bases_PCT	96.24	96.22	96.22	96.39	96.22
Mismatched_Bases_PCT	0.72	0.73	0.73	0.73	0.73
Median_Insert_Size	492	492	492	492	492
Percent Bases >20x	98.02	98.04	98.04	98.04	98.04
Average Coverage	39.56	39.61	39.61	39.61	39.61
Chimeric_Rate	2.51	2.6	2.6	2.6	2.6
