# Supplementary material for: Functional equivalence of genome sequencing analysis pipelines enables harmonized variant calling across human genetics projects
Source: Nat Commun. 2018 Oct 2;9:4038. doi: 10.1038/s41467-018-06159-4 (PMC6168605; doi:10.1038/s41467-018-06159-4)
Supplement: Supplementary file 2 — Peer Review File [file 41467_2018_6159_MOESM2_ESM.pdf]

REVIEWERS' COMMENTS:

Reviewer #1 (Remarks to the Author):

The authors have answered my reviews.

The french plan is not published but there is a comprehensive document with a stable looking URL

http://solidarites-sante.gouv.fr/IMG/pdf/genomic_medicine_france_ 2025.pdf

I think this is potentially worth citing or another, non US, non UK project.

Reviewer #2 (Remarks to the Author):

The authors were responsive to the previous criticism. The paper is good and strong which is at the level of Nat Communications.

Response to reviewers (referees' comments in blue, our response in black):
REVIEWERS' COMMENTS:

Reviewer #1 (Remarks to the Author):

The authors have answered my reviews.

The french plan is not published but there is a comprehensive document with a stable looking URL

http://solidarites-sante.gouv.fr/IMG/pdf/genomic_medicine_france_2025.pdf

I think this is potentially worth citing or another, non US, non UK project.

Thank you for the reference. We have added it.

Reviewer #2 (Remarks to the Author):

The authors were responsive to the previous criticism. The paper is good and strong which is at the level of Nat Communications.

Thank you for your time.
